# Pharmacological inhibition of the NLRP3 inflammasome attenuates kidney apoptosis, fibrosis, and injury in Dahl salt-sensitive rats

**DOI:** 10.1007/s10157-024-02567-7

**Published:** 2024-11-22

**Authors:** Yue Wang, Yuhang Wu, Jiayu Ren, Ying Wang, Imran Perwaiz, Hongtong Su, Jing Li, Peng Qu

**Affiliations:** Institute of Heart and Vessel Diseases, The Second Hospital Affiliated of Dalian Medical University, Dalian, 116000 China

**Keywords:** Kidney injury, MCC950, NLRP3 inflammasome, Rats, Salt-sensitive hypertension

## Abstract

**Background:**

Salt-sensitive hypertension (SSH) is the most severe form of hypertension, and the presence of NLRP3 inflammasome plays a crucial role in its pathogenesis. Although MCC950 has shown therapeutic potential for hypertension and kidney injury, its mechanism of action remains unclear.

**Methods:**

Dahl salt-sensitive (SS) rats and their salt-tolerant aptamer control SS-13^BN^ (BN) rats were randomly assigned to four groups: SS rats intraperitoneally administered physiological saline (SS + vehicle) or MCC950 (SS + MCC950), and BN rats intraperitoneally administered physiological saline (BN + vehicle) or MCC950 (BN + MCC950). All rats were given 2% saline for drinking and received intraperitoneal injections of physiological saline or MCC950 (5 mg/kg) every other day. Biomarkers such as serum creatinine, urinary protein, sodium retention, NLRP3 inflammasome, inflammation, apoptosis, fibrosis, sodium channels and histopathological changes in kidney injury were evaluated in blood, urine, and kidney tissues.

**Results:**

Compared with the SS + vehicle group, the SS + MCC950 group showed significantly lower blood pressure levels. Additionally, inhibition of NLRP3 inflammasome activation was observed along with reduced inflammation, apoptosis, fibrosis, and sodium retention in the kidneys.

**Conclusions:**

The findings suggest that pharmacological inhibition of the NLRP3 inflammasome reduces blood pressure in SS rats and alleviates related kidney injury by suppressing inflammation, apoptosis, fibrosis, and sodium retention.

**Supplementary Information:**

The online version contains supplementary material available at 10.1007/s10157-024-02567-7.

## Introduction

Hypertension is a major risk factor for cardiovascular disease including cerebrovascular events as well as chronic kidney disease [1]. Approximately 30–50% of hypertensive patients exhibit an increase in blood pressure due to salt sensitivity [2–5], with this figure rising to 75% in China [4, 6]. SSH (Salt-sensitive hypertension) poses a significant threat as it increases the risk of end-stage renal disease and mortality [5, 7–10]. When patients with salt sensitive hypertension consume a certain amount of salt, they need higher systemic blood pressure than that in normal subjects to excrete the salt from their kidneys due to shifted pressure-diuresis and natriuresis curves [4].

The NLRP3 inflammasome (NLR family, pyrin domain containing 3) is a well-studied sensor that triggers inflammation and plays a crucial role in the occurrence and development of various chronic diseases. In situations such as pathogen infection, stress, or cell destruction, the NLRP3 inflammasome receives dangerous signals and initiates an inflammatory cascade. The development of hypertension and its associated kidney injury is partly dependent on the presence of functional NLRP3 inflammasome, which consists of NLRP3, transfer protein ASC (apoptosis-associated speck-like protein containing CARD), and Caspase-1 [11, 12]. Once assembled, the NLRP3 inflammasome activates Caspase-1 to transform inactive pro-IL (Interleukin)-1β and pro-IL-18 into mature pro-inflammatory mediators IL-1β and IL-18 respectively, leading to a cascade inflammatory response [13–16]. Therefore, investigating the role of the NLRP3 inflammasome in hypertension and kidney injury could provide valuable insights.

MCC950 is a highly selective small molecule effective inhibitor of NLRP3. It is an adiarylsulfonylurea-containing compound with high efficiency in selectively inhibiting the oligomerization and activation of NLRP3 inflammasome [17–19]. MCC950 has been shown to play an important role in many disease treatment studies; for example preventing renal fibrosis in a model of crystalline nephropathy [20], reducing the development of atherosclerotic lesions [21], and alleviating steroid-resistant asthma [22]. Recent studies have demonstrated that inhibition of NLRP3 in 1 K/DOCA (Desoxycorticosterone-acetate) /salt-induced hypertensive mice can lower blood pressure, reduce kidney injury, and improve kidney function, however it should be noted that this study performed unilateral nephrectomy on mice and an additional large dose of DOCA was added [23], which may not accurately represent SSH model animals with dual kidneys. The Dahl salt-sensitive (SS) rat model exhibits proteinuria and kidney damage associated with elevated arterial pressure after a high salt diet, which closely resembles the pathophysiology observed in humans. This includes renal cortical fibrosis and glomerulosclerosis consistent with hypertensive subjects, making it an ideal experimental model for studying SSH and kidney injury in vivo [24–26].

Therefore, we utilized SS rats to investigate the efficacy of MCC950 in the development of hypertensive nephropathy.

## Materials and methods

### Animals and treatments

Adult male Dahl Salt-Sensitive (SS) rats and its salt-tolerant aptamer control SS-13^BN^ (BN) rats aging 6– 8 weeks (180–220 g) were given 2% saline instead of normal drinking water and were intraperitoneal injected saline or MCC950 (5 mg/kg) every other day for 8 weeks. The experimental procedures were approved by the Animal Ethics Committee of The Second Affiliated Hospital of Dalian Medical University (Ethics Approval 2015 No. 45).

### Measurement of sodium balance

Rats were individually housed in metabolic cages with external food containers and water bottles for the duration of the study. Saline intake and urine output were measured within a 24 h period. Daily sodium balance was calculated by subtracting sodium excretion from sodium intake [27].

### Statistical analysis

Quantitative data were presented as mean ± SD, Statistical analysis was performed using GraphPad Prism 8.0 software. The data were analyzed by the two-factorial ANOVA and a post-hoc Tukey’s multiple comparison test. p < 0.05 was considered statistically significant.

## Results

### Effect of MCC950 on the BP and kidney function in SS rats

**Fig. 1:**
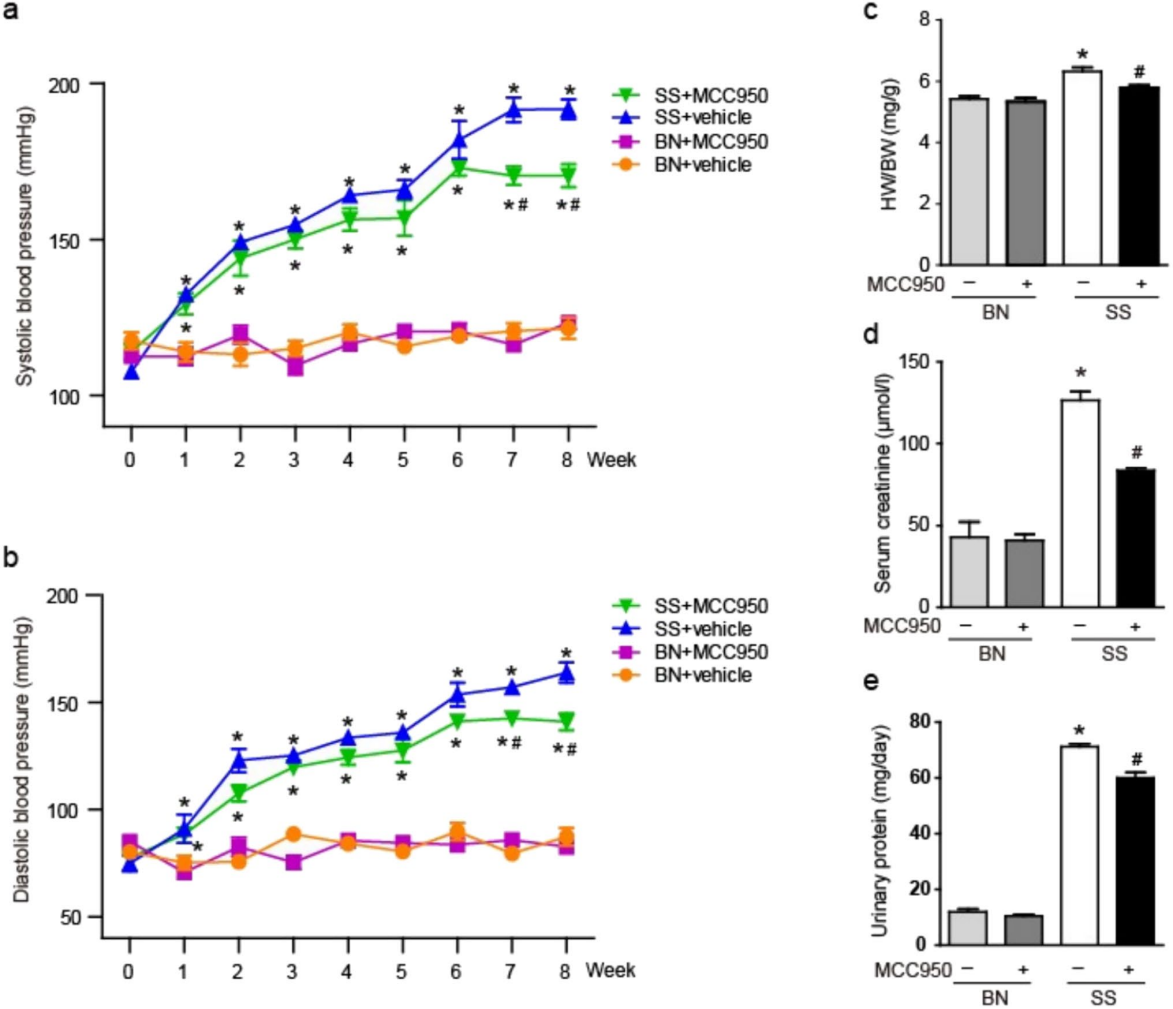
Effect of MCC950 on the BP and kidney function in SS rats. **a** Systolic blood pressure was measured via tail-cuff plethysmography at 10 min-intervals and plotted as an average over a 1 week period. **b** Diastolic blood pressure were measured. **c** The heart weight (HW) to body weight (BW) ratio was measured. **d** The volume of serum creatinine was measured by biological method. **e** The volume of urinary protein was measured by biological method. Values are expressed as mean ± SD (n = 4 ~ 6 in each group of rats). * P < 0.05 vs BN + vehicle and # P < 0.05 vs SS + vehicle

All rats completed the experiment under normal physiological activity. As shown in Table 1, after 8 weeks of high salt intervention, the average body weight of SS rats was slightly lower than that the BN rats, and there was no significant difference in body weight of rats after MCC950 intervention. The water intake of SS rats was lower than that the BN rats, and the water intake increased after MCC950 intervention. There was no significant difference in food intake between the two groups of rats. The systolic and diastolic blood pressure of SS rats rapidly increased compared with the BN rats, and MCC950 intervention resulted in a significant decrease in blood pressure (Fig. 1a, b). MCC950 can also reduce the increase in heart weight (HW) to body weight (BW) ratio caused by high salt in SS rats (Fig. 1c). To evaluate the extent of renal function impairment, we measured the serum creatinine levels in rats, and the results showed that the serum creatinine levels in the SS rats were significantly increased, while they decreased after treatment with MCC950 (Fig. 1d). The levels of urinary protein in SS rats were significantly increased, MCC950 can reduce the above indicators in SS rats (Fig. 1e).

**Table 1:** Physiological parameters in SS and BN rats following 8 weeks of a high salt diet with and without administration of MCC950

Group	BN + vehicle	BN + MCC950	SS + vehicle	SS + MCC950
Water intake (ml)	125.25 ± 0.50	123.75 ± 1.50	109.83 ± 4.79^*^	118.00 ± 2.83^#^
Food intake (g)	11.75 ± 1.26	12.5 ± 0.58	11.67 ± 1.03	11.33 ± 0.82
Body weight (BW) (g)	302.75 ± 11.18	305.75 ± 7.62	287.33 ± 7.71^*^	290.83 ± 11.07

The water intake, food intake and body weight were measured

Values are expressed as mean ± SD (n = 4 ~ 6 in each group of rats)

^*^ P < 0.05 vs BN + vehicle and # P < 0.05 vs SS + vehicle

### Effect of MCC950 on glomerular injury and inflammation in the kidney of SS rats

**Fig. 2:**
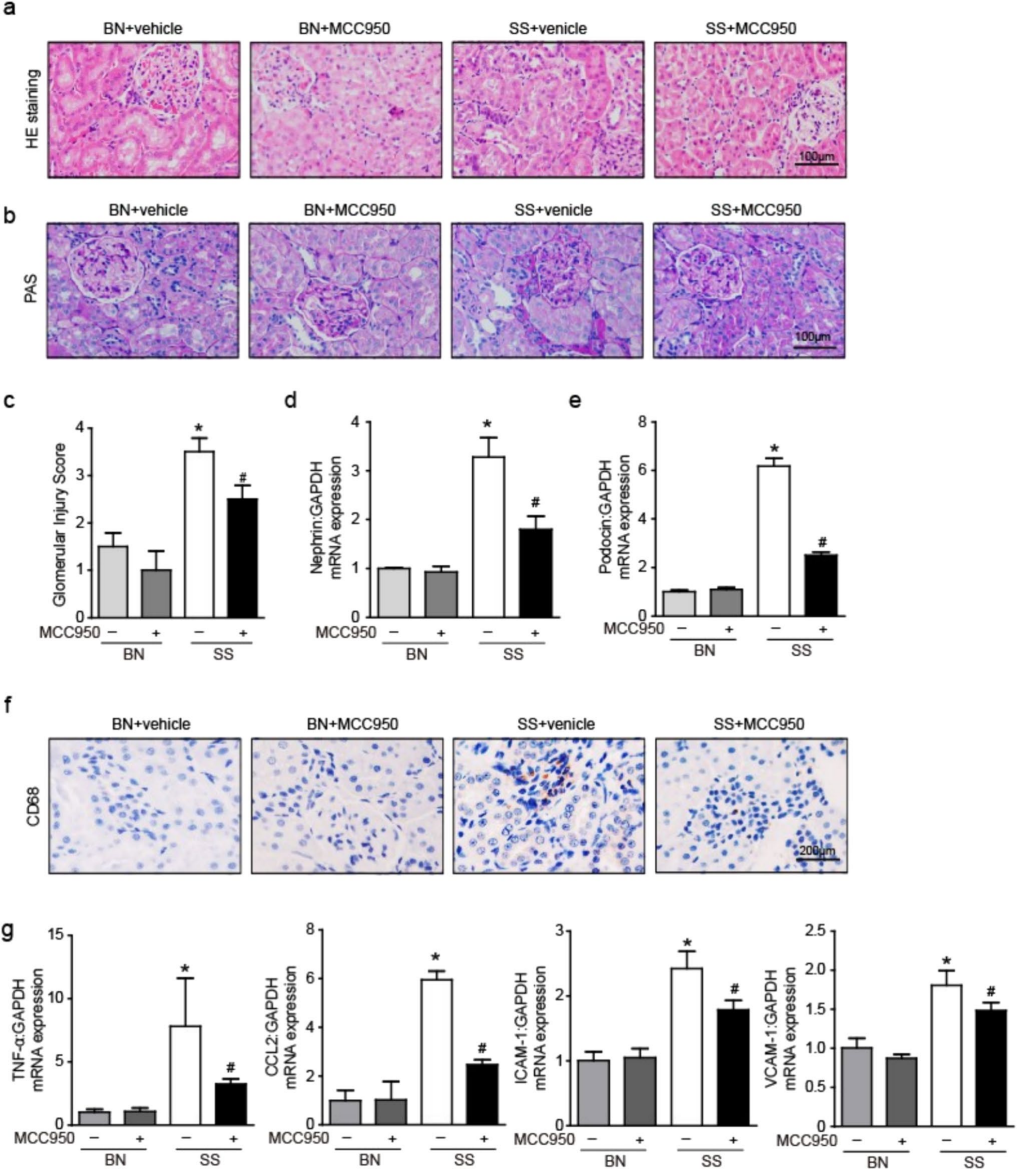
Effect of MCC950 on glomerular injury and inflammation in the kidney of SS rats. **a** HE staining of the kidney. **b** PAS staining of the kidney. **c** The degree of glomerular pathological damage score was performed in PAS staining. **d** The mRNA expression of the nephrin was measured. **e** The mRNA expression of the podocin was measured. **f** Representative images of kidney macrophage infiltration using anti-CD68 antibody. **g** The mRNA expression of the TNF-a, CCL2, ICAM-1 and VCAM-1 was measured. Messenger RNA expression was measured using the comparative Ct method against GAPDH expression. Values are expressed as mean ± SD (n = 4 ~ 6 in each group). * P < 0.05 vs BN + vehicle and # P < 0.05 vs SS + vehicle

We performed H&E staining and PAS staining to evaluate pathological changes. HE staining showed that the glomeruli of BN rats had a regular shape, complete structure, intact epithelial cell basement membrane, and no inflammatory cell infiltration in the renal interstitium. However, the glomerular structure of SS rats is more disordered, with blurred glomerular boundaries, enlarged tubular lumens, inflammatory cell infiltration, and thickening of the basement membrane. After treatment with MCC950, renal structural disorder was reversed and inflammatory cell infiltration was significantly reduced (Fig. 2a). In addition, PAS staining showed that compared with BN rats, SS rats exhibited thickening of the glomerular basement membrane, increased matrix in the mesangial area, widened mesangial tissue, segmental sclerosis, and increased glomerular injury score (Fig. 2b, c); We detected the mRNA expression of nephrin and podocin, which are related to glomerular injury, and found that the SS rats showed an increase compared to the BN rats **(**Fig. 2d, e**)**. After MCC950 intervention, the above changes were improved in SS rats **(**Fig. 2a–e**)**. Therefore, MCC950 treatment effectively reduced renal pathological damage in SS rats.

Kidney immunohistochemical analysis showed that high salt induced an increase in the accumulation of anti-CD68 expression positive cells in the renal interstitium, and MCC950 could inhibit the above phenomenon **(**Fig. 2f**)**. Chemokines and adhesion molecules are important mediators of leukocyte transport to tissues, causing leukocytes to move into kidneys. Similarly, an increase in the expression of several other pro-inflammatory genes including tumor necrosis factor-α (TNF-α), intercellular cell adhesion molecule-1 (ICAM-1), vascular cell adhesion molecule 1 (VCAM-1) and chemokine C–C motif ligand 2 (CCL2) was observed in SS rats. Treatment with MCC950 reduced the expression of these genes in SS rats **(**Fig. 2g**)**.

**Fig. 3:**
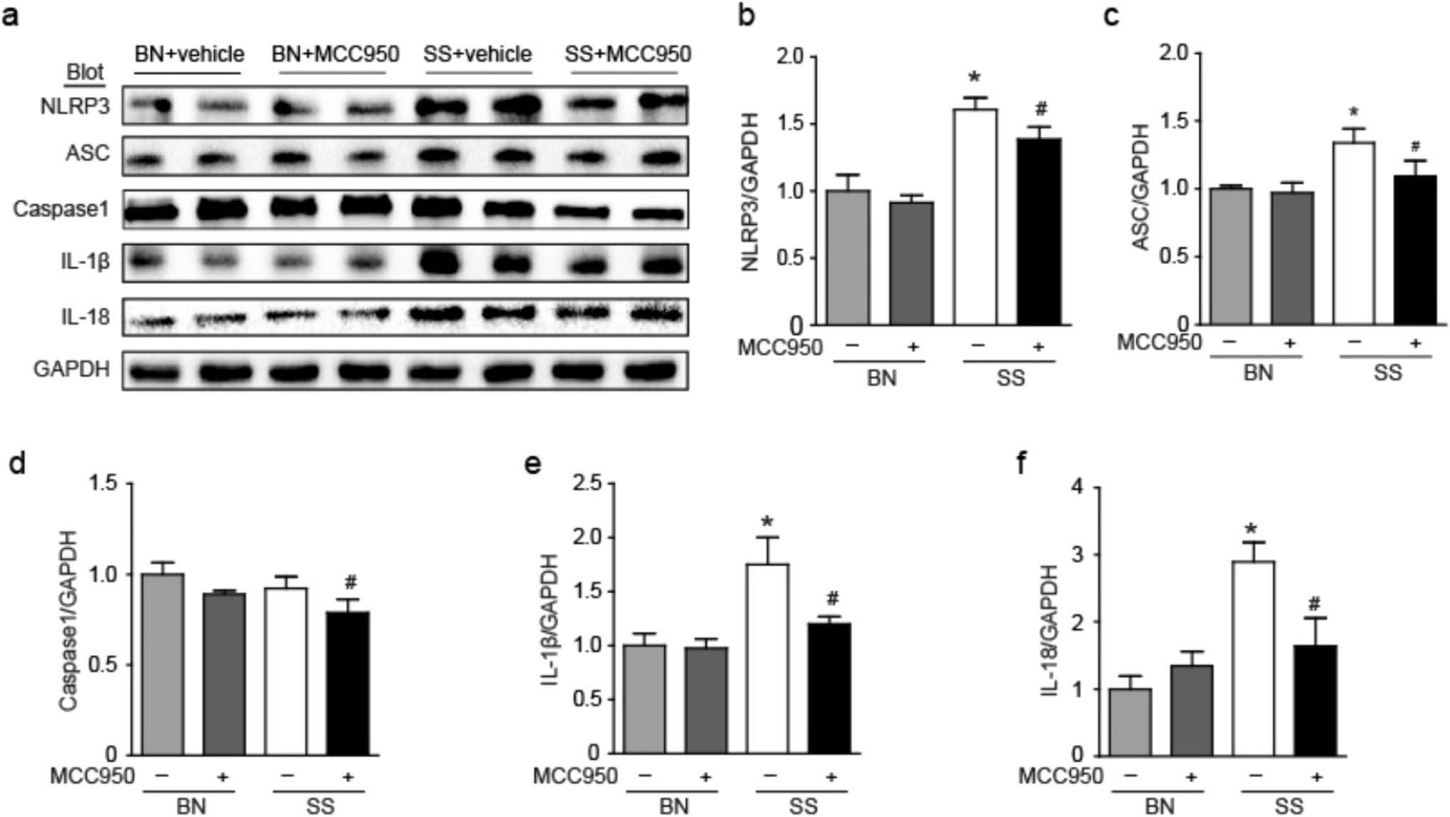
Effect of MCC950 on NLRP3 inflammasome in the kidney of SS rats. **a** Renal protein expression of the NLRP3, ASC, caspase-1, IL-1β, and IL-18 in rats was examined by WB. **b-f** Protein expression was measured using the comparation against GAPDH expression. Values are expressed as mean ± SD (n = 4 ~ 6 in each group of rats). * P < 0.05 vs BN + vehicle and # P < 0.05 vs SS + vehicle

We detected the expression of NLRP3 inflammasome in SSH kidneys through WB analysis, found that the expression of NLRP3, ASC, Caspase-1, IL-1β, and IL-18 was increased in SS rats, which was decreased by MCC950 treatment **(**Fig. 3a–f**)**. This hypertension model is associated with inflammation in the kidney, and the NLRP3/IL1β/IL-18 signaling system is involved.

### Effect of MCC950 on fibrosis in the kidney of SS rats

**Fig. 4:**
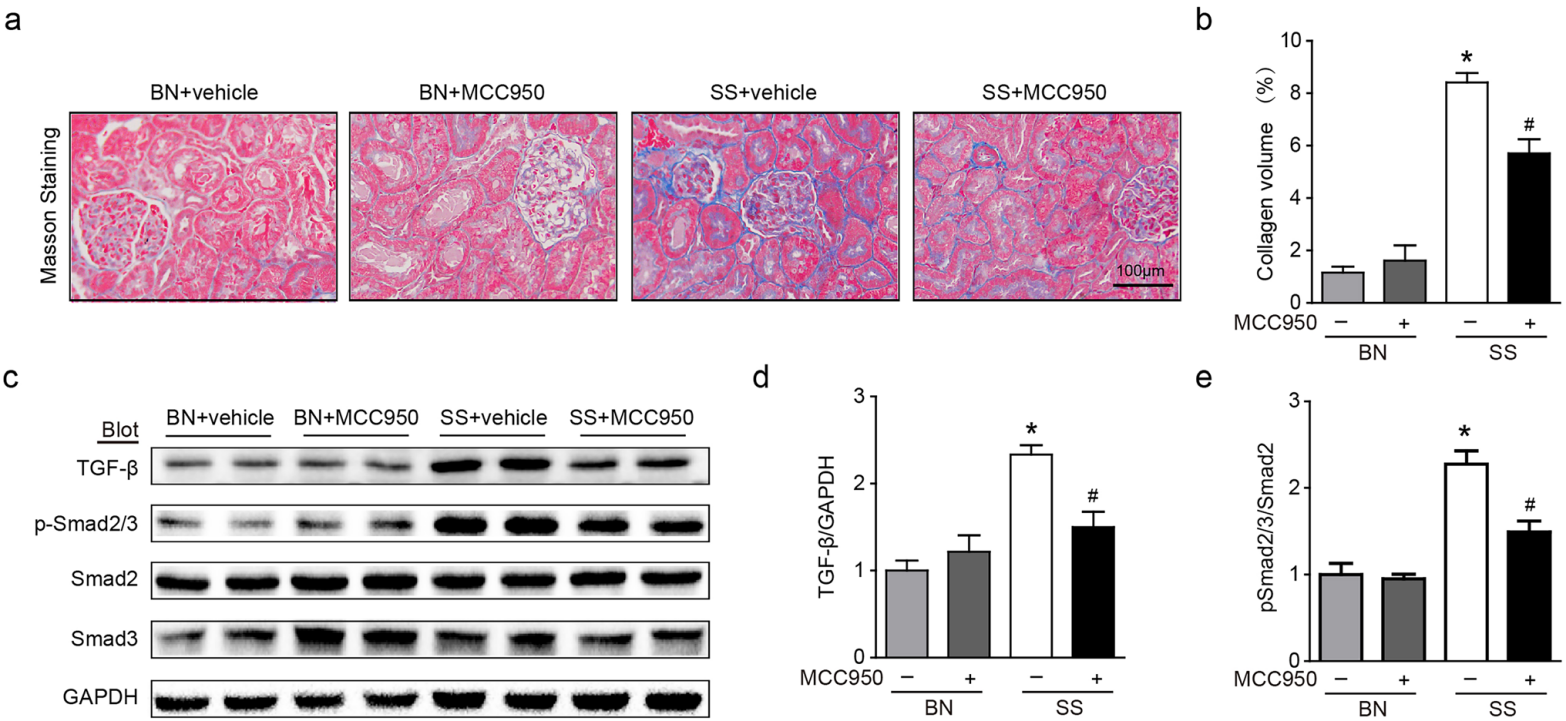
Effect of MCC950 on fibrosis in the kidney of SS rats. **a** Masson staining of the kidney. **b** Collagen volume was measured in each group. **c** Renal protein expression of the TGF-β and p-Smad2/3 in rats was examined by WB. **d** Protein expression of TGF-β was measured using the comparison against GAPDH expression. **e** Protein expression of p-Smad2/3 was measured using the comparison against GAPDH expression. Values are expressed as mean ± SD (n = 4 ~ 6 in each group of rats). * P < 0.05 vs BN + vehicle and # P < 0.05 vs SS + vehicle

Masson staining showed significant collagen deposition in the kidney of SS rats induced by high salt compared to the BN rats, while MCC950 treatment can reduce collagen deposition in SS rats **(**Fig. 4a , b**)**. The WB experiment also reached a similar conclusion, compared to the BN rats, the SS rats had higher expression of TGF-β and p-Smad2/3, and MCC950 treatment could significantly reduce the expression of TGF-β/Smad2/3 signaling pathway in SS rats **(**Fig. 4c–e**)**.

### Effect of MCC950 on cell apoptosis in the kidney of SS rats

**Fig. 5:**
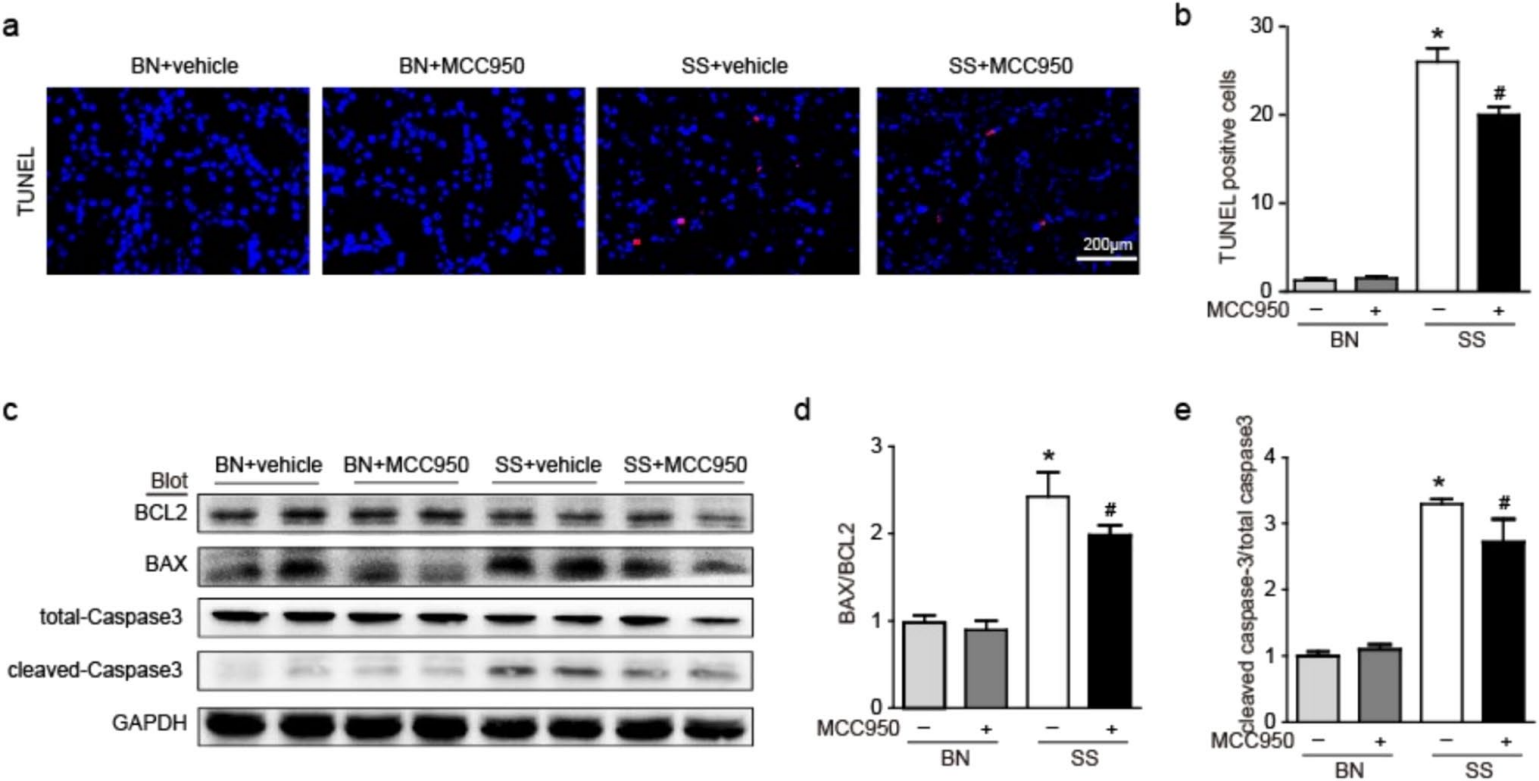
Effect of MCC950 on cell apoptosis in the kidney of SS rats. **a** TUNEL staining showed the apoptosis positive cell of renal tissue in rats. **b** Quantitative analysis of TUNEL positive cells in each group. **c** Renal protein expression of the cleaved-caspase3, total-caspase3, BAX and BCL2 in rats was examined by WB. **d** Protein expression of BAX was measured using the comparison against BCL2 expression. **e** Protein expression of cleaved-caspase3 was measured using the comparison against total-caspase3 expression. Values are expressed as mean ± SD (n = 4 ~ 6 in each group of rats). * P < 0.05 vs BN + vehicle and # P < 0.05 vs SS + vehicle

A TUNEL assay was performed to examine the effects of MCC950 on BN and SS rats. The number of TUNEL-positive cells was significantly higher in SS rats than in BN rats, and fewer positive cells were recorded in the SS + MCC950 group than in the SS group **(**Fig. 5a, b**)**. Western blot results demonstrated that the levels of cleaved Caspase-3/total Caspase-3 and BAX/BCL2 significant increase in SS rats’ kidney tissues compared to the BN rats, and were remarkably reduced after pretreatment with MCC950 **(**Fig. 5**)**. These results implied that cleaved Caspase-3, BCL2 and BAX may be involved in pathophysiological processes related to MCC950 alleviating effects in SSH kidney apoptosis.

### Effect of MCC950 on sodium balance and sodium channels in the kidney of SS rats

**Fig. 6:**
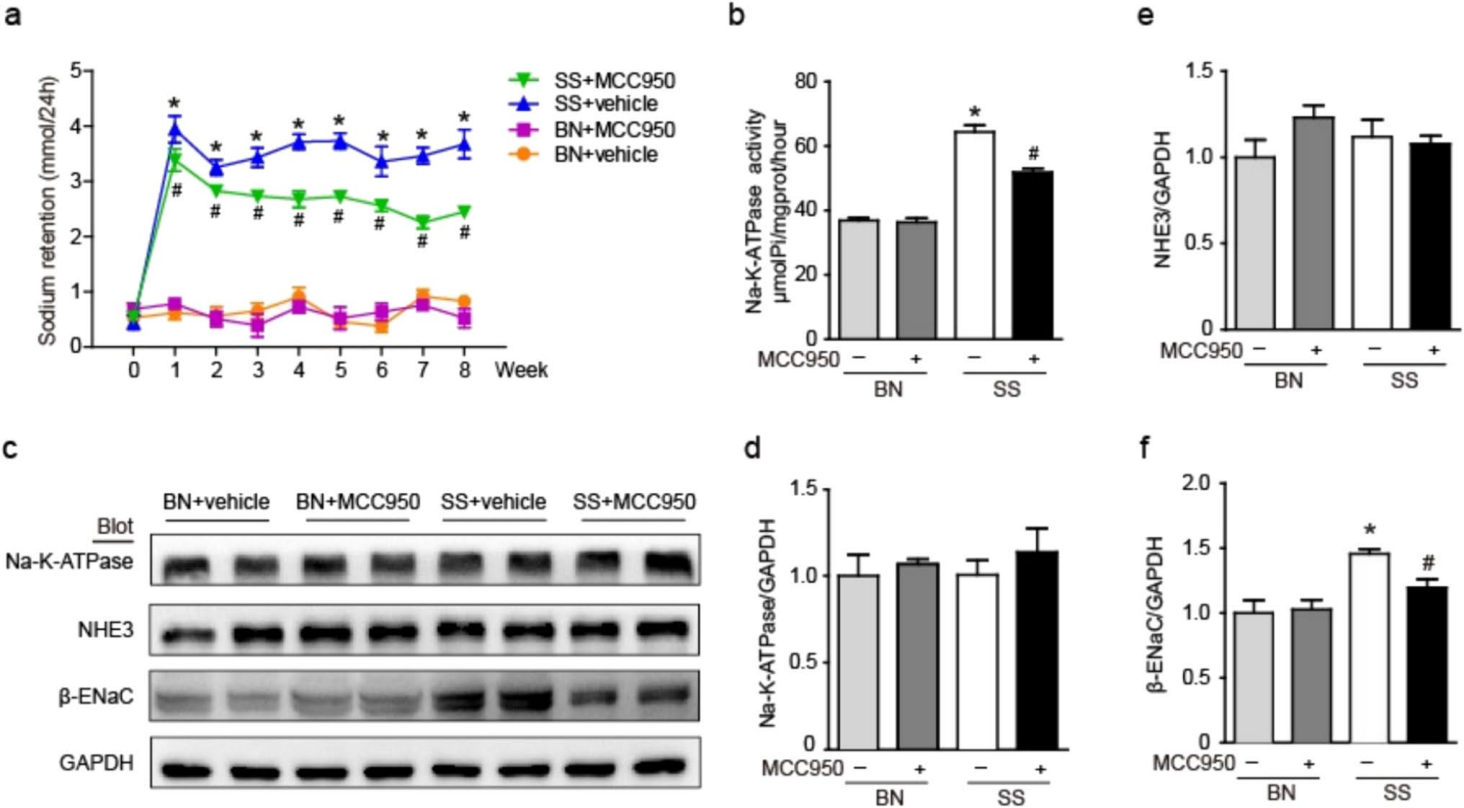
Effect of MCC950 on sodium balance and sodium channels in the kidney of SS rats. **a** 24 h sodium balance. **b** Na–K-ATPase activity in the renal was measured in rats. **c** Renal protein expression of the Na–K-ATPase, NHE3 and β-ENaC in rats was examined by WB. **d-f** Protein expression of Na–K-ATPase, NHE3 and β-ENaC were measured using the comparison against GAPDH expression. Values are expressed as mean ± SD (n = 4 ~ 6 in each group of rats). * P < 0.05 vs BN + vehicle and # P < 0.05 vs SS + vehicle

Compared to BN rats, SS rats had higher levels of 24 h sodium balance, and MCC950 treatment can improve the 24 h sodium balance in SS rats (Fig. 6a). In order to explore the mechanism of MCC950 affecting sodium retention, we detected renal Na–K-ATPase activity and found that compared to BN rats, SS rats maintained higher levels of Na–K-ATPase activity on a high salt diet, while MCC950 appropriately reduced the level of Na–K-ATPase activity (Fig. 6b). We conducted further testing on the protein expression of sodium channels in the kidney by WB. We found that there was no significant change in the protein expression of Na–K-ATPase and NHE3 among the four groups. In contrast, compared with BN rats, the renal protein expression of β-ENaC in SS rats were higher, and MCC950 treatment could reduce this abnormal phenomenon (Fig. 6c–f). These results implied that MCC950 treatment reduced sodium retention in SS rats.

## Discussion

SSH is the most severe form of hypertension, posing a higher risk of end-stage renal disease and mortality, and presenting a significant public health concern globally. The new finding of this study is that administration of MCC950 in SS rats can alleviate blood pressure, inflammation, apoptosis, fibrosis, and sodium retention in the kidney, as well as improve kidney function.

SSH induced kidney injury is a chronic, low-grade inflammatory disease [28]. The continuous inflammatory infiltration of the kidneys leads to the accumulation of activated fibroblasts and excessive production of extracellular matrix proteins (ECM), resulting in the destruction of renal parenchyma and gradual loss of renal function, which is the main pathogenesis of hypertensive renal fibrosis. TGF-β participates in various aspects of renal fibrosis through the TGF-β-Smads pathway. Previous studies have shown that targeted inhibition of the TGF-β signaling pathway can prevent kidney injury in SS rats [29]. Our study found that the expression of the TGF -β/Smad2/3 signaling pathway increased in the kidneys of SS rats, and administration of MCC950 significantly reduced renal interstitial collagen deposition and the expression of the TGF-β/Smad2/3 signaling pathway.

Hypertension causes podocyte damage and subsequent apoptosis, disrupting the final filtering barrier of the glomerulus and leading to glomerulosclerosis. The degree of podocyte injury and apoptosis is considered to be the main prognostic factors affecting hypertensive kidney damage. Alleviating podocyte apoptosis is of great significance for the treatment of hypertensive kidney damage. Previous studies have found that Ang II activates NLRP3 inflammasomes in podocytes, while NLRP3 silencing can attenuate Ang II induced podocyte apoptosis [30]. In the SS rat model, it was also found that high salt can lead to apoptosis of podocytes and renal tubules, while inhibiting apoptosis can alleviate kidney damage in SSH [31, 32]. The results of this study indicate that the increase in TUNEL positive cells in the kidneys of SS rats induced by a high salt diet was attenuated by MCC950. In addition, WB results showed that MCC950 treatment increased the expression of BCL2 in the kidneys of SS rats and decreased the expression of BAX and cleaved Caspase-3.

Recently, NLRP3 inflammasome has emerged as a promising therapeutic target for combating hypertension. Previous studies have found that hypertension is closely related to increased expression of renal adhesion molecules and pro-inflammatory cytokines, as well as the accumulation of inflammatory T cells and macrophages in the kidneys. Transgenic mouse models have demonstrated that the development of hypertension and its associated renal inflammation is at least partially dependent on the presence of functional NLRP3 inflammasomes [33, 34]. MCC950 has a targeted inhibitory effect on NLRP3 inflammasome, we found that MCC950 can reduce renal inflammatory infiltration in SS rats. This may be one of the mechanisms by which MCC950 lowers blood pressure. At the same time, the infiltrating immune cells around renal blood vessels and tubules may serve as a local source of bioactive molecules mediating vascular constriction, increasing sodium reabsorption in tubules, and promoting sodium retention—thereby exacerbating SSH [28]. Inhibiting the activation of NLRP3 inflammasome is crucial for improving kidney inflammation and lowering blood pressure. Previous studies have indicated abnormalities in the renal Na–K-ATPase sodium pump in SS rats leading to impaired excretion at high NaCl intake, which in turn affects systemic blood pressure. β-epithelial sodium channel (ENaC) was also found to be involved in renal sodium retention in SSH [35, 36]. The activation of ENaC by inflammatory factors such as TNF-α, IL-1β, and IL-6 accumulated in the kidneys has been confirmed [37, 38]. Therefore, inhibiting the activation of NLRP3 inflammasome is crucial for improving renal inflammation and suppressing abnormal renal sodium ion pumping. This study confirmed that the sodium retention of SS rats was obvious, which was improved after MCC950 treatment. Compared with BN rats, the renal Na–K-ATPase of SS rats remained highly active under high salt diet, and its activity decreased after MCC950 treatment. However, there was no significant difference in the protein expression of Na–K-ATPase among the groups. We further examined and found that the expression of β-ENaC remained at a high level and was inhibited by MCC950. This may be due to the fact that Na–K-ATPase mainly participates in the development of SSH by regulating Na^+^ transport efficiency, and the specific mechanism still needs further clarification, which is also a limitation of this study. In addition to reducing renal inflammatory infiltration, inhibiting the activity and expression of sodium channels and improving sodium retention may be one of the other mechanisms of MCC950 in reducing blood pressure.

In conclusion, our study demonstrates that inhibition of NLRP3 inflammasome can protect SS rats from the effects of inflammation, apoptosis, fibrosis, sodium retention and renal dysfunction related to the development of SSH. Therefore, MCC950 as a pharmacological inhibitor of NLRP3 inflammasome may represent a promising and feasible treatment strategy to combat SSH and prevent the development of kidney injury.

## Supplementary Information

Below is the link to the electronic supplementary material.

Supplementary file1 (DOCX 27 KB)

## Data Availability

No data was used for the research described in the article.
