# Supplementary material for: Pharmacological inhibition of the NLRP3 inflammasome attenuates kidney apoptosis, fibrosis, and injury in Dahl salt-sensitive rats
Source: Clin Exp Nephrol. 2024 Nov 22;29(1):113–22. doi: 10.1007/s10157-024-02567-7 (PMC11807026; doi:10.1007/s10157-024-02567-7)
Supplement: Supplementary file 1 — Supplementary file1 (DOCX 27 KB) [file 10157_2024_2567_MOESM1_ESM.docx]

**Supplementary Material**

**Pharmacological inhibition of the NLRP3 inflammasome attenuates kidney apoptosis, fibrosis, and injury in Dahl salt-sensitive rats**

**1. Materials and methods**

1.1. Drug

MCC950(S8930, Sellect, China) was suspended in stroke-physiological saline solution prior to use.

1.2. Animals and treatments

Adult male Dahl Salt-Sensitive (SS) rats and its salt-tolerant aptamer control SS-13^BN^ (BN) rats aging 6 to 8 weeks (180-220g) were purchased from Vitalriver Laboratory Animal Technology Company and placed in Dalian Medical University SPF Animal Experiment Center. The rats were placed and fed in the laboratory under controlled settings at room temperature (22°C) with 12 h of light and 12 h of darkness. The experimental procedures were approved by the Animal Ethics Committee of The Second Affiliated Hospital of Dalian Medical University (Ethics Approval 2015 No. 45).

These two genetic strains of animals were fed with SPF grade experimental breeding rat feed (Xietongshengwu, China) containing 20% protein, 10% water, 8% crudeash, 5% fibre, 4% fat, 1.8% calcium, and 0.2% sodium chloride. Rats were given 2% saline instead of normal drinking water and randomly divided into physiological saline or MCC950 intraperitoneal injection treatment groups. This resulted in four cohorts: SS rats receiving intraperitoneal injection of physiological saline (SS+vehicle) or MCC950 (SS+MCC950), and BN rats receiving intraperitoneal injection of physiological saline (BN+vehicle) or MCC950 (BN+MCC950). Inject either physiological saline or MCC950 (5mg/kg) intraperitoneally every other day. In the 8th week, rats were anesthetized with tetrabromoethanol (Sigma, reserve concentration 20mg/ml, dose calculated as 0.5g/kg body weight), and blood samples were immediately collected. Kidney tissue was rapidly frozen in liquid nitrogen or fixed with 4% neutral formaldehyde fixative, and then embedded in paraffin. There was no rat died before the end of this study.

1.3. Blood pressure measurement

BP (Blood pressure) was monitored weekly via tail-cuff plethysmography by WT1205000, Kent Science, USA.

1.4. Measurement of sodium balance

Metabolic balance studies were conducted before high salt intake and on days 7, 14, 21, 28, 35, 42, 49, and 56. Rats were individually housed in metabolic cages with external food containers and water bottles for the duration of the study. Saline intake and urine output were measured within a 24-hour period. Daily sodium balance was calculated by subtracting sodium excretion from sodium intake[1].

1.5. Urine examination

Rats were individually housed in metabolic cages for 24h to collect their 24h urine. And then the urinary protein was detected (Urine protein test kit C035-2-1, Nanjing Jiancheng Institute of Biological Engineering, China).

1.6. Na-K-ATPase activity assay

Proteins were extracted from renal cortex samples and the Na-K-ATPase activity was measured by a commercially available Na-K-ATPase assay kit (BC0065, Solarbio, Beijing, China) according to the manufacturer's protocol.

1.7. Histological analysis

The kidney was cut in half transversely, fixed with 4% neutral formaldehyde fixative, embedded in paraffin and cut into 4μm, and then stained with HE (haematoxylin-eosin) and PAS (Periodic acid-Schiff) for light microscopy, and stained with Masson trichrome for collagen. Glomerulus morphology was observed under HE and PAS staining. After PAS staining, at least 40 glomeruli per group were randomly selected to assess the severity of glomerulus injury, and the grading was as follows: 0 = normal, Grade 1 < 25%, Grade 2 = 25–50%, Grade 3 = 50–75%, and Grade 4 = 75–100%. Assessment criteria include the thickening of the glomerular basement membrane, the glomerular mesangial cells proliferation, mesangial matrix deposition, segmental sclerosis and podocyte injury[2].

1.8. Immunohistochemistry (IHC)

Prepare paraffin-embedded sections, followed by baking, deparaffinization, and ethanol hydration. Incubate CD68 primary antibody (Abcam, ab125212, 1:50) at 4℃ overnight, followed by incubation with secondary antibody (SolelyBio, China) the next day. Add a color reagent for reaction, then counterstain with hematoxylin and differentiate with 1% hydrochloric acid alcohol. Wash thoroughly and return to blue with ammonia solution. Finally, dehydrate and seal the sections, and observe the expression of the target protein in renal tissue under different treatments under microscopy.

1.9. TUNEL staining

TUNEL staining was performed using the one-step TUNEL cell apoptosis detection kit (KeyGEN BioTECH, JiangSu, China) following the manufacturer's protocol. Briefly, paraffin slices were dewaxed using conventional methods, permeated with sodium citrate in a microwave for 8 mins at high temperature, and then incubated with the TUNEL reaction mixture at 37 °C in a humid atmosphere for 60 mins. Every fifth section of each sample was collected and TUNEL-positive cells were counted under a fluorescence microscope (DM6B Thunder, Leica, Germany) at a magnification of 400 times.

1.10. Western Blot (WB) analysis

Total proteins from renal cortices were extracted with ice-cold RIPA lysis buffer (Beyotime, China), and protein concentrations were measured by BCA Protein Assay Kit (Cooler, China). An equal amount of proteins was separated by SDS-PAGE and transferred to PVDF membranes (Merck Millipore, Germany). The membranes were blocked in 5% nonfat milk at room temperature for 1 hr and then incubated overnight at 4°C with primary antibodies as follows: NLRP3 (30109-1-AP, proteintech, China), ASC (67494-1-Ig), Caspase-1 (22915-1-AP), IL-18 (10663-1-AP), IL-1β (16806-1-AP), BAX (50599-2-Ig), BCL2(68103-1-Ig), β-ENaC (14134-1-AP), p-Smad2/3 (8828S, CST, USA), Smad2 (5339S), Smad3 (9513S), total Caspase-3 (14220S), cleaved Caspase-3 (9664S), Na-K-ATPase (3010S), GAPDH (2118S) and NHE3 (DF9937, Affinity, China). After incubation with the appropriate secondary antibodies (7074S, 58802S, CST, USA) at room temperature for 1 hr, target bands were detected by high-sensitivity chemiluminescent fluid (Tanon, China). Semi quantitative analysis was measured using Image J software.

1.11. RNA extraction and real-time qPCR

Total RNA was extracted from renal cortices using TRIzol (SolelyBio, China). Reverse transcription was performed with the Evo M-MLV reverse transcription reagent kit (AG11705, AG, China), and 1 μg of RNA was reverse transcribed into cDNA. The rat gene sequence was obtained from NCBI, and the primers and probes were designed using Primer Premier 5.0 and synthesized by General Biosystems (AnHui, China). The upstream primer for TNF-α was CAAGAGCCCTTGCCCTAAGG, and the downstream primer was CGGACTCCGTGATGTCTAAGTACTT. For CCl2, the upstream primer was GGCCTGTTGTTCACAGTTGCT, and the downstream primer was CCTGCTGCTGGTGATTCTCTT. Icam-1 had an upstream primer of GTCTGTCAAACGGGAGATGAATG and a downstream primer of CCCGCAATGATCAGTACCAA. Vcam-1's upstream primer was AGGCTGGAATTAGCAAAAAATCAG, while its downstream primer was TTATCCATATTTCGGGCACACTT. Real-time qPCR analysis was performed using SYBR Green reagent on a QuantStudio™ 5 real-time PCR System (AG11701, AG, China) following the manufacturer’s instructions. The PCR conditions consisted of an initial denaturation at 95°C for 30s followed by 40 cycles of denaturation at 95°C for 5s and annealing/extension at 60°C for 30s. Relative quantitative expression levels were calculated using the 2^−ΔΔCt^ method with each group examined in triplicate.

**References:**

[1] R.D. Wainford, C.Y. Carmichael, C.L. Pascale, J.T. Kuwabara, Galphai2-protein-mediated signal transduction: central nervous system molecular mechanism countering the development of sodium-dependent hypertension, Hypertension, 65 (1) (2015) 178-186.

[2] X. Li, Z. Liu, Z. He, X. Wang, R. Li, J. Wang, et al., Acteoside protects podocyte against apoptosis through regulating AKT/GSK-3beta signaling pathway in db/db mice, Bmc Endocr Disord, 23 (1) (2023) 230.
